# The future of immunotherapy for diffuse large B‐cell lymphoma

**DOI:** 10.1002/ijc.35156

**Published:** 2024-09-25

**Authors:** Johannes Duell, Jason Westin

**Affiliations:** ^1^ Department of Internal Medicine 2 University Hospital of Würzburg Würzburg Germany; ^2^ Department of Lymphoma and Myeloma MD Anderson Cancer Center Houston Texas USA

**Keywords:** diffuse large B‐cell lymphoma, DLBCL

## Abstract

With the introduction of anti‐CD19 chimeric antigen receptor (CAR) T‐cell (CAR T) therapies, bispecific CD3/CD20 antibodies and anti‐CD19 antibodies, immunotherapy continues to transform the treatment of diffuse large B‐cell lymphoma (DLBCL). A number of novel immunotherapeutic strategies are under investigation to build upon current clinical benefit and offer further options to those patients who cannot tolerate conventional intensive therapies due to their age and/or state of health. Alongside immunotherapies that leverage the adaptive immune response, natural killer (NK) cell and myeloid cell‐engaging therapies can utilize the innate immune system. Monoclonal antibodies engineered for greater recognition by the patient's immune system can enhance antitumor cytotoxic mechanisms mediated by NK cells and macrophages. In addition, CAR technology is extending into NK cells and macrophages and investigational immune checkpoint inhibitors targeting macrophage/myeloid cell checkpoints via the CD47/SIRPα axis are in development. Regimens that engage both innate and adaptive immune responses may help to overcome resistance to current immunotherapies. Furthermore, combinations of immunotherapy and oncogenic pathway inhibitors to reprogram the immunosuppressive tumor microenvironment of DLBCL may also potentiate antitumor responses. As immunotherapy treatment options continue to expand, both in the first‐line setting and further lines of therapy, understanding how to harness these immunotherapies and the potential for combination approaches will be important for the development of future DLBCL treatment approaches.

## Graphical abstract

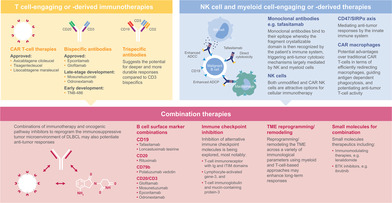

## 1 BACKGROUND

First‐line R‐CHOP (rituximab plus cyclophosphamide, doxorubicin, vincristine, and prednisone) and similar regimens are curative in 60%–70% of patients with diffuse large B‐cell lymphoma (DLBCL), with 30%–40% of patients experiencing relapse or a primary refractory disease course.^1^ However, some patients are unable to tolerate the R‐CHOP regimen due to older age, being unfit/frail, and/or having comorbidities.^2^, ^3^ Furthermore, some patients are at high risk of relapse/refractory (R/R) disease following first‐line R‐CHOP due to higher International Prognostic Index (IPI) score^4^ or high‐grade lymphoma classification (MYC plus BCL2 and/or BCL6 rearrangements, also known as double or triple hit lymphomas).^5^ Effective treatment options for these patients are currently lacking.

A number of treatment strategies have been evaluated to improve the efficacy of first‐line therapy, or provide novel options for patients who are frail/elderly. Intensified chemotherapy approaches have not improved upon the R‐CHOP first‐line standard of care and have been associated with increased toxicity.^6^, ^7^, ^8^ In older and/or unfit patients with DLBCL, approaches to improve tolerability of anthracycline‐based chemotherapy may compromise efficacy. Rituximab with dose‐reduced CHOP (R‐miniCHOP)^9^ and ofatumumab (anti‐CD20)‐miniCHOP^10^ in patients older than 80 years with DLBCL and who have a good performance status improved tolerability and provided an effective treatment option, albeit with response and survival rates lower than in younger patients with DLBCL. The addition of targeted therapies such as Bruton's tyrosine kinase inhibitors (BTKis) and BCL2 inhibitors to first‐line R‐CHOP have similarly met with limited success, failing to show significant efficacy improvements and are associated with increased toxicity.^11^, ^12^, ^13^, ^14^ The use of targeted chemotherapy in the form of antibody–drug conjugates (ADCs) has proven a more successful strategy. The Phase III POLARIX study evaluating a modified regimen of R‐CHOP (pola‐R‐CHP), in which vincristine was replaced with polatuzumab vedotin (an ADC targeting CD79b) in patients with previously untreated intermediate‐risk or high‐risk DLBCL, showed a statistically significant slightly lower risk of disease progression, relapse or death compared to R‐CHOP.^15^

Immunomodulation with lenalidomide, which has been shown to block tumor cell proliferation and angiogenesis, and stimulate T‐ and NK cell‐mediated cytotoxicity, has also been evaluated as a strategy to improve current DLBCL treatment options, but has met with mixed results. Lenalidomide in combination with R‐CHOP (also known as R2‐CHOP) had numerically greater progression‐free survival (PFS) and overall survival (OS) compared to R‐CHOP; however, the study was not powered adequately for definitive comparisons.^16^ The Phase III ROBUST study, in which a lower dose of lenalidomide was used, did not meet the primary endpoint of PFS but did report a nonsignificant positive trend in 2‐year PFS rate in a subgroup analysis of patients with high‐risk disease (IPI scores ≥3 and advanced disease stages).^17^ In elderly and unfit/frail patients, a chemotherapy‐free option of lenalidomide plus rituximab has been assessed as an alternative to standard first‐line anthracycline‐containing regimens. Although the Phase II ReRI study did not meet its primary efficacy endpoint, the observed clinical activity warrants further exploration of this combination.^18^

**FIGURE 1:**
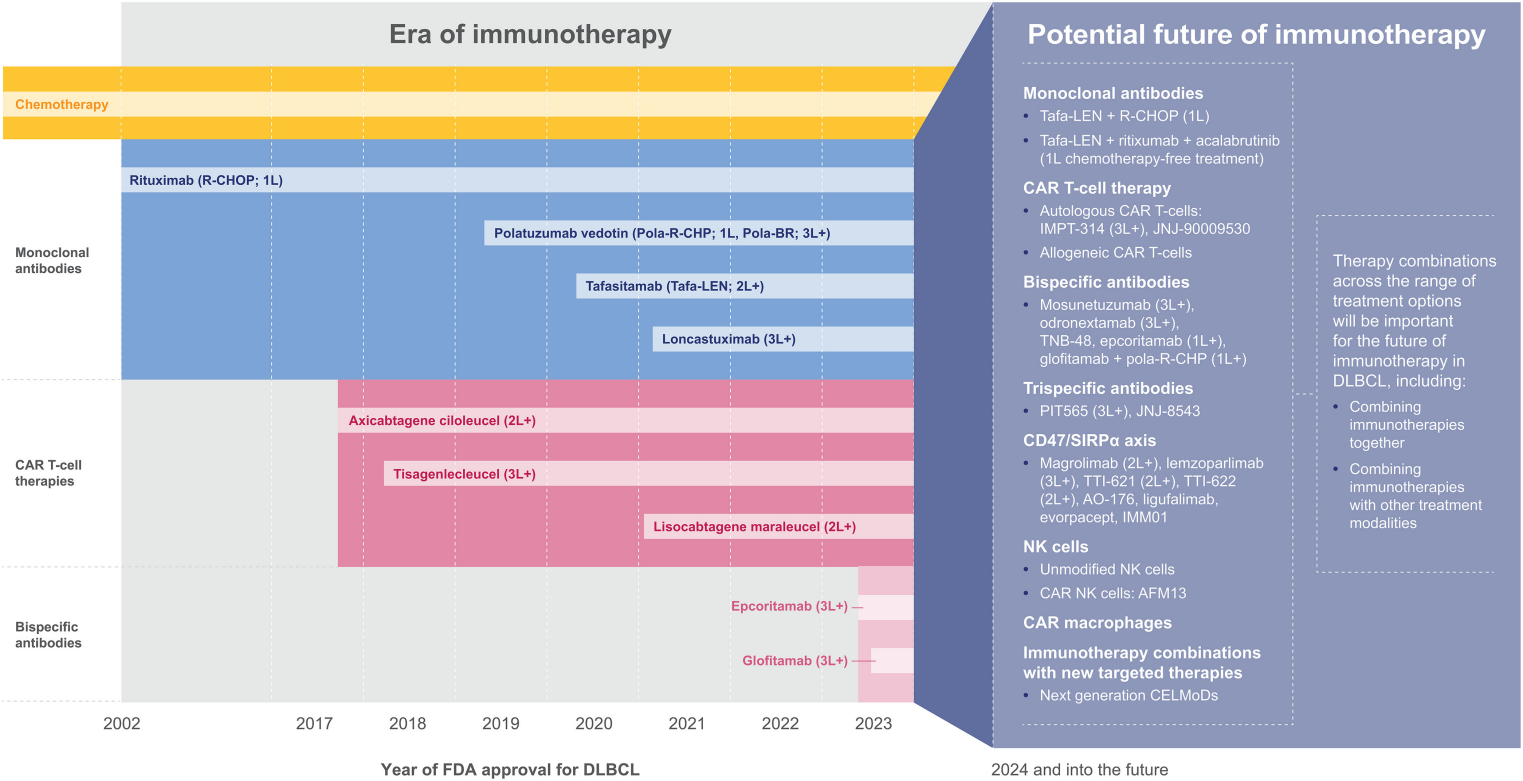
A timeline of the era of immunotherapy for DLBCL. CAR T‐cell, chimeric antigen receptor T‐cell; CELMoD, cereblon E3 ligase modulator; CHOP, cyclophosphamide, doxorubicin, vincristine, and prednisone; DLBCL, diffuse large B‐cell lymphoma; FDA, US Food and Drug Administration; NK, natural killer.

**FIGURE 2:**
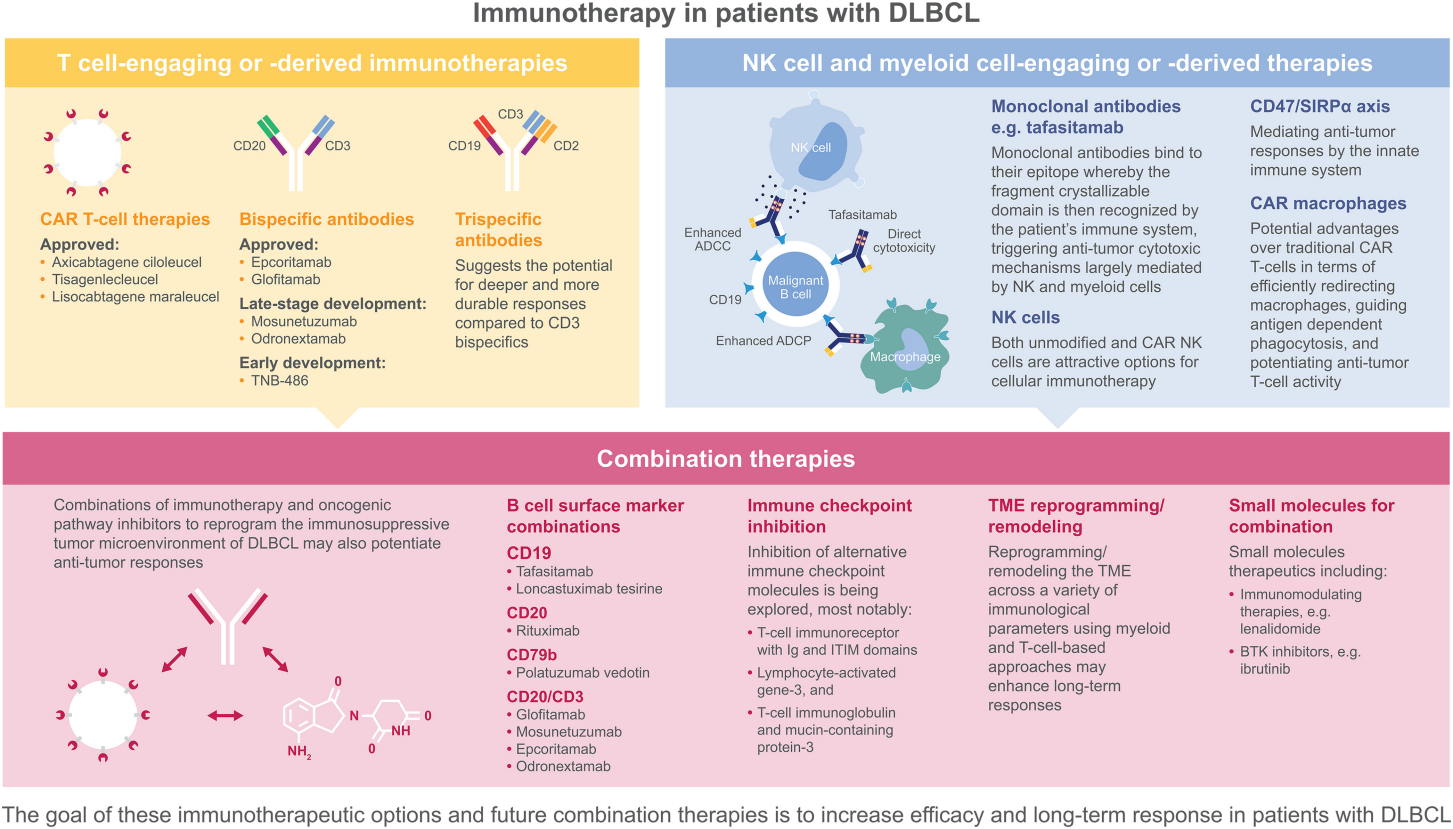
Current and future immunotherapeutic treatments for DLBCL. ADCC, antibody‐dependent cellular cytotoxicity; ADCP, antibody‐dependent cellular phagocytosis; BTK, Bruton's tyrosine kinase; CAR T‐cell, chimeric antigen receptor T‐cell; DLBCL, diffuse large B‐cell lymphoma; ITIM, immunoreceptor tyrosine‐based inhibitory motif; NK, natural killer; TME, tumor microenvironment.

Immunotherapy has revolutionized the treatment of R/R DLBCL through the introduction of anti‐CD19 antibodies, anti‐CD19 CAR T‐cell (CAR T) therapies, and bispecific CD3/CD20 antibodies (Figure 1). While these approaches have shown significant clinical benefit in R/R DLBCL and hold promise for patients with treatment‐naïve and high‐risk DLBCL, there are still challenges to overcome. Looking to the future of DLBCL management, utilizing novel immunotherapies that harness the immune system will be critical to addressing the current unmet need. The objective of this review is to outline the most relevant immune pathways and promising agents that target them, and explore the potential synergies offered by combination approaches (Figure 2).

## 2 IMMUNOTHERAPIES UTILIZE A RANGE OF APPROACHES TO SUPPORT THE IMMUNE SYSTEM TO ELIMINATE CANCER CELLS

Both myeloid cells of the innate immune system and lymphocytes of the adaptive immune response have a critical role in immune surveillance and control of B‐cell lymphomas. T cells primarily act either as cytotoxic CD8+ T cells attacking cells or as CD4 helper T cells (CD4+ T cells) supporting B‐cell antigen production. T cells are classically activated by an interaction between the T‐cell receptor (TCR) and a major histocompatibility complex (MHC)‐bound antigen on antigen‐presenting cells,^19^ resulting in a complex cascade of activation pathways. The key antitumor effector mechanism mediated by CD8+ T cells that is exploited by several immunotherapeutic strategies is perforin/granzyme‐mediated cell death. Mobilization and activation of CD4+ T cells is also crucial to mounting an effective antitumor response in DLBCL, as patients with DLBCL and >20% infiltrating CD4+ T cells have better relapse‐free survival and OS.^20^, ^21^ However, 50% of DLBCL cases lack cell surface expression of MHC Class I^22^ with loss of MHC Class II expression also reported,^23^ both of which compromise effective tumor immunosurveillance.

NK cells of the innate immune system target cancer cells through the detection of MHC Class I molecule downregulation or stress markers.^24^ Activation of their cytotoxicity, cytokine production, and proliferation is mediated via a balance of signals from activating and inhibitory receptors, such as FcγRIII (CD16), NKG2D, killer cell immunoglobulin‐like receptors and immune checkpoint molecules.^24^ Several immunotherapeutic strategies exploit the potent cytotoxic function of NK cells in an MHC‐independent manner through FcγRIII (CD16) engagement and subsequent antibody‐dependent cellular cytotoxicity (ADCC). Myeloid cells, comprising macrophages, dendritic cells, monocytes, and granulocytes, function as key antigen‐presenting cells and play a pivotal part in tumor clearance through macrophage‐mediated antibody‐dependent cellular phagocytosis (ADCP) following chemotherapy and immunotherapy. Myeloid cells represent a significant proportion of the tumor immune microenvironment in DLBCL^25^ and can create an immune suppressive or stimulatory environment that positively or negatively influences cancer progression and metastasis.^26^ Modulating myeloid cell functions to create a stimulatory, antitumor environment may augment the efficacy of current and novel treatment options and provides a new avenue for the development of immunotherapeutic strategies.

Several novel therapies based on T‐cell, NK‐cell or myeloid cell‐engaging or ‐derived therapies are under development. Thus far, these emerging immunotherapies are primarily under investigation in the R/R DLBCL disease setting, with the hope of a widening scope to also improve first‐line options.

### 2.1 T cell‐engaging or ‐derived immunotherapies

#### 2.1.1 CAR T‐cell therapies

CAR T‐cell therapies are derived from blood cells modified in vitro using viral transduction to express a CAR on the cell surface, which can target a tumor antigen. The most common current version of CAR is an anti‐CD19 single‐chain variable fragment linked to an intracellular signaling domain (usually CD3ζ), resulting in MHC‐independent T‐cell activation upon binding CD19 on the surface of malignant B cells and triggering cytotoxicity.^27^ In DLBCL, a number of autologous CAR T‐cell therapies have been approved in the third‐line R/R disease setting: axicabtagene ciloleucel (axi‐cel; ZUMA‐1^28^, ^29^), tisagenlecleucel (tisa‐cel, JULIET^30^), and lisocabtagene maraleucel (liso‐cel, TRANSCEND‐NHL‐001^31^). These third‐line CAR T‐cell therapies have shown robust and long‐term responses in DLBCL, and demonstrated similar efficacy in several real‐world studies.^32^ More recently, the use of second‐line CAR T‐cell therapy has been approved following data from the ZUMA‐7 and TRANSFORM/PILOT studies.^33^, ^34^, ^35^ The high response rates and the durability of the responses have led to the initiation of a number of studies in treatment‐naïve patients with high‐risk LBCL (ZUMA‐12^36^; ZUMA‐23^37^).

Practical considerations with CAR T‐cell therapy include treatment center resources and potential immune‐mediated side effects.^32^ The requirement for autologous T cells for the manufacture of the treatment product may also limit patient eligibility as many patients are heavily pretreated and may have unfit T cells.^38^ Due to the CAR T‐cell manufacturing time, bridging therapy may be required; however, the use of bridging therapy has shown to be associated with worsened outcomes, likely due in part to the reasons why bridging is required such as aggressive disease.^39^ CAR T‐cell therapies are also associated with toxicities such as cytokine‐release syndrome (CRS) and neurotoxicities.^40^, ^41^

Continued development in the CAR T‐cell therapy space is focused on improvement of treatment efficacy. Due to the immunosuppressive tumor microenvironment (TME) in patients with solid tumors, a barrier is created, limiting the long‐term efficacy of CAR T‐cell therapy in these patients, resulting in further cancer progression.^42^, ^43^, ^44^ One method under consideration to address this issue is the creation of armored CAR T cells, which are engineered to express proteins alongside the CAR in an effort to reduce immunosuppression and improve antitumor efficacy.^42^, ^43^, ^44^ Armored CAR T‐cell therapy includes TRUCK (T cells Redirected toward Universal Cytokine Killing) CARs, cytokine modulating CARs and antibody‐secreting CARs. Another emerging approach, potentially with more immediate relevance to patients with DLBCL, uses CAR T cells directed at multiple B‐cell antigens, rather than just CD19, such as CD20 and CD22, which has the potential to improve response by preventing antigen escape.^45^ Investigational candidate IMPT‐314, a CD19/20 bispecific CAR T‐cell therapy, is in Phase I/II development in patients with R/R B‐cell non‐Hodgkin lymphoma (R/R B‐NHL; NCT05826535).^46^

“Off‐the‐shelf” allogeneic CAR T cells have several potential advantages over autologous CAR T cells, including shorter manufacturing time, greater manufacturing scalability, and avoidance of poor T‐cell “fitness” from heavily pretreated patients. Despite their potential advantages, allogeneic products may also present specific issues such as short persistence due to rapid elimination by the immune system, along with other risks such as graft‐versus‐host disease (GvHD) and graft rejection, which can be minimized with genomic editing during allogeneic CAR T‐cell manufacture.^47^ A number of allogeneic CAR T cells are under clinical investigation.^48^ The most common adverse events associated with allogeneic CAR T cells are mild‐to‐moderate cytopenias, CRS and neurotoxicity, with negligible incidences of GvHD reported.^49^

#### 2.1.2 Bispecific and trispecific antibodies

Bispecific antibodies are a promising T‐cell‐mediated approach which use the specificity of monoclonal antibodies to engage the patient's own T cells to kill malignant B cells. Engineered by combining two different monoclonal antigen‐specific binding regions from different antibodies into one molecule, they simultaneously target both a tumor cell and a T‐cell epitope.^50^

In DLBCL, the majority of bispecific antibodies currently in advanced clinical development target CD20 on B cells and CD3 of the TCR complex, bypassing MHC/TCR binding and resulting in T‐cell engagement.^51^ CD3–CD20 bispecific antibodies epcoritamab and glofitamab recently received regulatory approval in the United States, and Canada and Europe, respectively, for R/R DLBCL, with mosunetuzumab and odronextamab in advanced clinical development in patients with R/R disease. Bispecific antibodies in combination with CHOP+/−R are also under evaluation as first‐line therapy.^50^, ^51^, ^52^ TNB‐486 is a novel CD3–CD19 bispecific antibody in Phase I development in R/R DLBCL.^53^

As with CAR T‐cell therapy, CRS has been shown to be associated with bispecific antibodies, but generally to a lower incidence and severity than with CAR T.^51^ To further mitigate potential for CRS, “step‐up dosing,” subcutaneous formulations, or the use of a cytoreductive anti‐CD20 antibody prior to administration of the bispecific therapy are being evaluated.^54^

Taking the concept of multitargeting introduced with bispecific antibodies a step further, trispecific antibodies are at an early stage of investigation, with PIT565 demonstrating simultaneous engagement of CD19 on tumor cells, CD3 and CD2 on T cells leading to redirected T‐cell cytotoxicity toward CD19+ malignant B cells. Preclinical data have suggested the potential for deeper and more durable responses compared to CD3 bispecifics and a Phase I investigation is underway in patients with R/R B‐NHL and R/R B‐cell acute lymphoblastic leukemia.^55^ A further trispecific antibody, JNJ‐8543 (JNJ‐80948543), is also being investigated in a Phase I trial across a range of indications, including DLBCL.^56^

#### 2.1.3 Beyond T‐cell‐engaging or ‐derived immunotherapies

While long‐term efficacy data for bispecific antibodies are just beginning to emerge,^57^ recently reported long‐term efficacy data from studies of CAR T‐cell therapies show that ~60%–70% of patients experience a relapse within 2–3 years of treatment initiation.^29^, ^30^, ^58^ These results suggest that additional therapeutic options are needed. Combination therapy utilizing the innate immune system via NK cell and myeloid cell‐engaging or ‐derived therapies may improve upon current immunotherapies.

### 2.2 NK cell and myeloid cell‐engaging or ‐derived therapies

#### 2.2.1 Monoclonal antibodies

Rituximab, the CD20‐targeting monoclonal antibody introduced the significant role for monoclonal antibody immunotherapy in B‐cell NHL. Monoclonal antibodies bind to their epitope whereby the fragment crystallizable (Fc) domain is then recognized by the patient's immune system, triggering antitumor cytotoxic mechanisms (ADCC, ADCP, and complement‐dependent cytotoxicity) largely mediated by NK and myeloid cells.

In treatment‐naïve patients with DLBCL, ~15%–20% have low CD20 expressing tumors.^59^, ^60^ CD19, however, is ubiquitously expressed throughout the B‐cell maturation process and is broadly conserved across many B‐cell malignancies.^59^, ^60^, ^61^, ^62^ Tafasitamab is an anti‐CD19, humanized, monoclonal antibody with an engineered Fc region for increased affinity for FcγRIII, leading to increased ADCC and ADCP by NK cells and macrophages.^63^, ^64^ ADCC and ADCP are further enhanced when tafasitamab is used in combination with lenalidomide, which induces FcγRIII expression on NK cells, allowing for a greater degree of activation following tafasitamab binding.^65^ Tafasitamab plus lenalidomide has shown an overall response rate of almost 60% and a duration of response of 43.9 months in patients with R/R DLBCL in the L‐MIND study, with a 5‐year analysis demonstrating long‐term durable responses (median duration of response not reached).^66^, ^67^

#### 2.2.2 NK cells

Therapeutic monoclonal antibody approaches in DLBCL rely on the engagement of NK cells via FcγRIII (CD16) to facilitate ADCC of targeted cells. Due to their intrinsic cytotoxic capacity and low potential for autoreactivity inherent with T‐cell approaches, NK cells themselves are attractive options for cellular immunotherapy.

NK cells that have been expanded and activated, although otherwise unmodified, do not require antigen presentation (unlike T‐cell‐based therapies) when used as an adoptive cell therapy.^68^ Autologous and haploidentical NK cells have been investigated alone and in combination with various other therapies in several hematological cancers. From a safety perspective, allogeneic NK cells benefit from limited reported potential toxicity issues compared to allogeneic CAR T cells, including GVHD.

CAR NK cells could offer several advantages over CAR T cells, such as rapid generation from multiple allogeneic sources offering an “off‐the‐shelf” option for patients with heavily pretreated or rapidly progressing disease, reduced risk of alloreactivity inherent to NK cells and a superior safety profile in terms of little to no reported incidence of CRS or neurotoxicity.^69^, ^70^, ^71^ A recent Phase I/II trial confirmed the preliminary efficacy and tolerability of CAR NK cells derived from cord blood in heavily pretreated patients with CD19‐positive lymphoid cancers including two patients with refractory DLBCL.^69^ Clinical trials are ongoing to further evaluate the safety and efficacy of this treatment approach. Interestingly, a novel approach utilizing cytokine‐induced cord blood‐derived NK cells precomplexed with the CD30/CD16 bispecific antibody, AFM13, demonstrated CAR NK cell‐like features in preclinical studies and clinical activity in a proof‐of‐concept study in patients with R/R CD30+ Hodgkin's or non‐Hodgkin's lymphoma.^72^

#### 2.2.3 CD47/SIRPα axis

The CD47/SIRPα axis is an important checkpoint, mediating antitumor responses by the innate immune system. CD47 is ubiquitously expressed and binds to SIRPα, an inhibitory receptor expressed on the surface of macrophages, to inhibit macrophage recognition and subsequent phagocytosis.^73^, ^74^ In DLBCL, overexpression of CD47 was associated with worse clinical outcomes in patients with DLBCL treated with CHOP±R.^75^ Exploitation of the CD47/SIRPα axis by blocking CD47‐SIRPα signaling represents a promising therapeutic strategy with several Phase I/II studies underway evaluating anti‐CD47 monoclonal antibodies (magrolimab [Hu5F9‐G4]; ligufalimab [AK117]; lemzoparlimab [TJC4]; AO‐176) or anti‐SIRPα Fc‐fusion proteins (evorpacept [ALX148]; TTI‐621; TTI‐622; IMM01).

#### 2.2.4 CAR macrophages

CAR technology has also extended to macrophages, wherein preclinical models suggest CAR macrophages have potential advantages over traditional CAR T cells in terms of efficiently redirecting macrophages, guiding antigen‐dependent phagocytosis, and potentiating antitumor T‐cell activity.^76^ CAR macrophages are currently only in clinical development in solid tumors.

## 3 POTENTIAL FOR NOVEL COMBINATION THERAPIES

### 3.1 B‐cell surface marker combinations

Targeting two or more B‐cell surface markers simultaneously may limit resistance via antigen loss.^59^, ^60^, ^77^ Targeting CD19 and CD20 simultaneously with tafasitamab and rituximab, supplemented by lenalidomide to enhance ADCC activity, in combination with CHOP has demonstrated promising clinical activity as first‐line therapy in the Phase Ib First‐MIND study, with no new adverse safety signals to what would be expected.^78^ First‐line tafasitamab and lenalidomide plus R‐CHOP is currently under further investigation in the ongoing, fully enrolled Phase III frontMIND study (NCT04824092). Glofitamab in combination with R‐CHOP or pola‐R‐CHP is being evaluated in treatment‐naïve patients with DLBCL (NCT04914741).^79^ In the R/R DLBCL setting, polatuzumab vedotin plus rituximab‐chemotherapy combinations have shown to be effective treatment combinations.^80^ The combination of polatuzumab vedotin and mosunetuzumab is being studied in the Phase III SUNMO trial in patients with R/R DLBCL.^81^

Enhanced NK cell‐mediated ADCC evident in preclinical data^65^ is utilized in L‐MIND with the combination of tafasitamab and lenalidomide^66^ illustrating the potential for immunomodulatory drugs to boost current monoclonal antibody therapy activity. TAK‐981, a small molecule inhibitor of the small ubiquitin‐like modifier (SUMO), brings in another synergistic approach, as a potent and selective inhibitor of SUMOylation. This reversible posttranslational modification results in an inflammatory response mediated by type 1 interferons, increased NK cell activation and M1 macrophage polarization.^82^ Combinations of tafasitamab and rituximab with TAK‐981 were found to markedly enhance ADCC and ADCP and augment antitumor activity compared to the respective monotherapies.^82^ Immunomodulation of the adaptive immune response is being evaluated with the combination of epcoritamab with lenalidomide as first‐line therapy for DLBCL.^83^

With the availability of an array of immunotherapy modalities targeting cell surface molecules, a broad range of combination approaches are now being assessed with the objective of developing chemotherapy‐free approaches. The Phase II Smart Stop study (NCT04978584) is assessing the combination of tafasitamab, rituximab, lenalidomide, and the BTKi acalabrutinib as an immunotherapy combination alone or with CHOP in patients with treatment‐naïve nongerminal center (non‐GCB) DLBCL.^84^ This trial aims to build on the proof‐of‐concept Smart Start trial (NCT02636322), which showed that the combination of rituximab, lenalidomide, and ibrutinib followed by sequential addition of chemotherapy led to high overall response rates and durable responses compared to historical outcomes,^85^ highlighting the feasibility of such an approach. Other chemotherapy‐free combination approaches with monoclonal antibodies, ADCs, and bispecific antibodies are being explored.^86^ Mosunetuzumab is being investigated as consolidation therapy and as monotherapy or in combination with polatuzumab vedotin in elderly/unfit patients with previously untreated DLBCL (NCT03677154).

A phase II study of mosunetuzumab, polatuzumab, tafasitamab, and lenalidomide in patients with relapsed B‐cell NHL is ongoing (NCT05615636).^87^ Interestingly, the sequence of the immunotherapy combinations may be a consideration. Preclinical data suggest the sequential use of tafasitamab followed by CD19‐targeted CAR T‐cell therapy in xenograft models ameliorated side effects of T‐cell overactivation and promoted antitumor activity of CAR T‐cell therapy.^88^, ^89^ Loncastuximab tesirine, an ADC targeting CD19, was being evaluated in combination with rituximab in a Phase II trial in previously untreated unfit/frail patients with DLBCL; however, the study was terminated due to adverse respiratory safety signals (LOTIS‐9; NCT05144009).^86^, ^90^, ^91^ Other potential treatment options include cereblon E3 ligase modulators (CELMoDs) that are being investigated in combination with immunotherapeutics. A phase I/II study investigating the CELMoD golcadomide alone and in combination with rituximab in patients with R/R DLBCL or R/R follicular lymphoma (FL) is currently underway (NCT03930953).^92^ Preliminary results have shown that golcadomide plus rituximab has a similar safety profile to golcadomide alone and has shown promising efficacy outcomes. Another CELMoD, avadomide, has completed a phase I trial in combination with rituximab in patients with R/R DLBCL or R/R FL, with positive safety outcomes and preliminary antitumor activity reported (NCT03283202).^93^ Other CELMoD candidates are under investigation, and may eventually be used in combination with immunotherapies; however, these agents are outside of the scope of the current review.

### 3.2 Immune checkpoint inhibition

A recent systematic review and meta‐analysis reported limited clinical benefit with immune checkpoint inhibitors (ICIs) targeting programmed cell death protein 1 (PD‐1) and its ligand (PD‐L1) or cytotoxic T‐lymphocyte‐associated protein 4 (CTLA‐4) as monotherapy in R/R DLBCL.^94^ Inhibition of alternative immune checkpoint molecules is being explored to enhance immunostimulatory and antitumor activity in DLBCL, most notably T‐cell immunoreceptor with Ig and ITIM domains (TIGIT), lymphocyte‐activated gene‐3 (LAG‐3), and T‐cell immunoglobulin and mucin‐containing protein‐3 (TIM‐3), all of which mediate suppression of T‐cell activation. Several early‐phase studies are underway to evaluate the blockade of these immune checkpoint molecules in DLBCL.^95^, ^96^, ^97^, ^98^, ^99^, ^100^

While most ICI combinations focus on T‐cell effector function, inhibitors of the macrophage/myeloid cell checkpoint via the CD47/SIRPα axis are also being studied as a combination partner to further enhance the efficacy of DLBCL immunotherapy. Magrolimab in combination with rituximab has shown promising activity with rapid and durable responses in a Phase Ib/II study in patients with R/R DLBCL.^101^, ^102^ Preclinical data show combining tafasitamab with an anti‐CD47 monoclonal antibody increases the ADCP activity of primary macrophages, resulting in enhanced antitumor activity compared to tafasitamab or anti‐CD47 monotherapies alone.^103^, ^104^ To further maximize the immunotherapy combination of tafasitamab plus lenalidomide, the addition of TTI‐622 is being studied in a Phase Ib/II study in patients with R/R DLBCL (NCT05626322). A similar study is ongoing for ALX148, rituximab, and lenalidomide for the treatment of indolent and aggressive B‐NHL (NCT05025800). The potential for a combined antagonism of CD47/SIRPα and PD‐1/PD‐L1 pathways is being evaluated in a Phase II study of TTI‐622 and TTI‐621 in combination with pembrolizumab for the treatment of R/R DLBCL (NCT05507541).

### 3.3 Tumor microenvironment reprogramming/remodeling

Tumor antigen heterogeneity, limited immune cell trafficking and infiltration, T‐cell exhaustion, MHC downregulation, and anti‐inflammatory cytokines are all key features of the “noninflamed” or immunosuppressive DLBCL tumor microenvironment (TME).^20^, ^105^, ^106^, ^107^ Reprogramming or remodeling the TME across a variety of immunological parameters using myeloid and T‐cell‐based approaches may potentially enhance long‐term responses.

#### 3.3.1 Enhancing immune cell infiltration

DLBCL is characterized by low immune cell infiltration, especially in the GCB subtype.^20^, ^107^, ^108^ The presence of tumor‐infiltrating lymphocytes is indicative of immune‐inflamed tumors and an active antitumor immune response.^107^ Tumor infiltration of CD4 T+ cells may shift the balance of suppressive and pro‐inflammatory cytokines toward a TME more conducive to infiltration of cytotoxic T cells or CAR T cells. NK cell and myeloid cell‐engaging or ‐derived therapies have the potential to produce pro‐inflammatory cytokines and chemokines to recruit other immune cells, such as CAR T cells and macrophages.^76^, ^109^, ^110^

#### 3.3.2 Switching to a pro‐inflammatory cytokine tumor microenvironment

A challenge for maintaining immunotherapy responses in DLBCL is the presence of anti‐inflammatory cytokines, such as TGF‐β, interleukin‐10 (IL‐10), and vascular endothelial growth factor (VEGF), principally produced by tumor‐associated macrophages (TAMs), myeloid‐derived suppressor cells, and cancer‐associated fibroblasts.^105^, ^107^ Modulating the cytokine environment through allogeneic or CAR NK cells, and potentially CAR macrophages, may enhance immunotherapy responses by promoting an immune‐inflamed TME to maintain immune cell activation.^76^, ^109^, ^110^

#### 3.3.3 Addressing T‐cell exhaustion

In addition to the immunosuppressive TME, chronic or persistent T‐cell exposure to tumor antigens is associated with marked changes in T‐activation and differentiation, leading to T‐cell “dysfunction” or “exhaustion”, defined by poor effector function, sustained expression of inhibitory receptors, and a transcriptional state different to that of functional effector or memory T cells.^111^, ^112^ In DLBCL, T‐cell exhaustion may be a mechanism for DLBCL progression after treatment with indirect T cell‐engaging immunotherapy, bispecific antibodies, or CAR T‐cell therapies, particularly if multiple T‐cell approaches are used.^111^ Expression of inhibitory checkpoint molecules, such as PD‐1, CTLA‐4, LAG‐3, TIGIT, and TIM‐3 are phenotypically characteristic of exhausted T cells.^38^, ^111^, ^113^, ^114^, ^115^ Variability in CAR T‐cell efficacy may be linked to heterogeneity in the cellular and molecular features of CAR T‐cell infusion products with the inclusion of T cells with exhausted characteristics, particularly high expression of LAG‐3 and TIM‐3.^38^ T cells may be reactivated or activation maintained with simultaneous or optimally sequenced approaches.

#### 3.3.4 Oncogenic signaling pathway inhibition within the TME

Molecular profiling of the TME identifies several genomic and oncological drivers that can be therapeutically targeted alongside immune cell strategies and lead to novel combinations.^116^ Targeting the B‐cell receptor and its intracellular signaling pathways presents a logical rationale for combination with immunotherapy. Indeed, data from the Smart Start and Smart Stop studies suggest the inclusion of BTKis with immunotherapy to be a promising approach. Inhibitors of the phosphatidylinositol 3 kinase (PI3K) signaling pathway can promote T‐cell activation and inhibit immunosuppressive immune cell populations.^116^ Bcl2 is a characteristic of GCB‐derived, high‐grade B‐cell lymphomas, and studies have already reported for venetoclax in DLBCL. However, as with BTKis and PI3K inhibitors, these studies have been conducted thus far in combination with chemoimmunotherapeutic regimens and are often associated with notable toxicities, probably underlying the biological complexity and requiring greater optimization for effective combination with immunotherapy.^117^ Aberrant epigenetic programming affecting the lymphoma TME has been reported, with epigenetic markers enriched in DLBCL, notably the GCB phenotype.^108^, ^118^, ^119^ EZH2‐activating mutations are associated with MHC downregulation in DLBCL.^120^ EZH2 inhibition with tazemetostat or tulmimetostat, in addition to targeting other epigenetic mechanisms with histone deacetylase inhibitors, may play a role in modulating the TME by restoring immune signaling.^119^ The hypoxic state of the DLBCL TME plays a fundamental role in immune evasion as hypoxia can induce the expression of immunosuppressive factors, such as VEGF, induce the expression of inhibitory immune checkpoints such as PD‐(L)1, LAG‐3, TIM‐3, CTLA‐4, and CD47^121^ and modulate pro‐tumorigenic TAMs.^122^ Combinations of immunotherapy and therapies targeting hypoxia inducible factor‐1α/VEGF, PI3K, or mitogen‐activated protein kinase signaling pathways may offer novel therapeutic approaches to overcome limitations and maximize antitumor immunity.

## 4 CONCLUSIONS

Advances in immunotherapy will continue to transform the future of DLBCL therapy. Combination therapy leveraging both the innate and adaptive immune systems simultaneously could overcome known immunotherapy “resistance” mechanisms to current T‐cell‐derived immunotherapies (e.g., loss of antigen, T‐cell exhaustion, immunosuppressive TME). Novel NK cell and myeloid cell‐engaging therapy combinations may further improve clinical benefit, not only in the R/R setting, but also in the first‐line setting where chemotherapy has not compromised the patient's immune system. Furthermore, chemotherapy‐free options offer treatments with more tolerable safety profiles for patients who are elderly or frail/unfit and unable to tolerate conventional intensive chemotherapies.

## AUTHOR CONTRIBUTIONS

**Johannes Duell:** Conceptualization; writing—review and editing. **Jason Westin:** Conceptualization; writing—review and editing.

## FUNDING INFORMATION

This work was supported by MorphoSys AG and Incyte Corporation.

## CONFLICT OF INTEREST STATEMENT

Johannes Duell declares: Nothing to disclose. Jason Westin declares: Research funding from: ADC Therapeutics, Allogene, BMS, Genentech, Janssen, Kite/Gilead, MorphoSys/Incyte, Novartis, Nurix; Consulting funding from: AbbVie, ADC Therapeutics, Allogene, BMS, Genentech, Genmab, Janssen, Kite/Gilead, MorphoSys/Incyte, Novartis, Nurix, and Pfizer.
